# High sensitivity detection of Hepatitis B virus RNA based on 3D-DNA nanomachine and protein nanopore sensing

**DOI:** 10.1186/s43556-025-00282-7

**Published:** 2025-08-08

**Authors:** Shixin Yan, Chuipeng Kong, Jiazhe Cheng, Zhuoyun Tang, Ke Sun, Shanchuan Chen, Minghan Li, Chengyan Tao, Yue Li, Yanhua Zhao, Chuanmin Tao, Jia Geng, Feng Li

**Affiliations:** 1 Department of Laboratory Medicine, State Key Laboratory of Biotherapy and Cancer Center, West China Hospital, Sichuan University and Collaborative Innovation Center, Chengdu, 610041 China; 2 Key Laboratory of Green Chemistry & Technology of Ministry of Education, College of Chemistry, Sichuan University, Chengdu, 610041 China; 3 Department of Laboratory Medicine, West China Hospital, Sichuan University, Chengdu, 610041 China

**Keywords:** 3D-DNA nanomachine, Nanopore sensing, Hepatitis B virus RNA, ssDNA, Clinical detection

## Abstract

Serum Hepatitis B virus (HBV) RNA serves as a non-invasive biomarker for monitoring chronic hepatitis B and guiding treatment decisions. However, current diagnostic tests relying on Reverse Transcription Quantitative Polymerase Chain Reaction (RT-qPCR) require centralized facilities and tedious operational steps, and there is currently no standardized HBV RNA detection method. Herein, we describe the rapid amplification-free detection of HBV RNA in clinical serum samples via a nanopore sensing method. Our method involves the design of a DNA nanomachine capable of translation of each target HBV RNA into a large amount of 7-nt single-strand DNA (ssDNA) as signal reporters that produce blockage features when passing through an alpha-hemolysin (α-HL) nanopore, the target can be quantified by identifying the frequency of this blockage occurrence. We demonstrate that this unique nanopore sensing feature not only distinguishes Signal Report (SR) DNA from interfering nucleic acids in clinical serum samples but also ensures a high assay sensitivity with a limit-of-detection (LOD) at 12.5 fM. The clinical potential of this method was validated through testing serum samples from 26 HBV-infected-patients and 7 healthy-participants. When compared with the RT-qPCR method, it showed only one false result, achieving an accuracy rate of 97.0%. Additionally, testing results for 18 cases during the treatment period demonstrated that our method’s conclusions on continued treatment and drug discontinuation were highly consistent with clinical diagnoses, with an accuracy rate of 100%. Therefore, the method developed in this study expands the clinical applications of 3D DNA nanomachine and nanopore sensing technology in clinical detection.

**Supplementary Information:**

The online version contains supplementary material available at 10.1186/s43556-025-00282-7.

## Introduction

Each year, approximately 800,000 people worldwide die from liver diseases caused by hepatitis B virus (HBV) infection. Despite the ability of current HBV vaccines to transiently suppress the virus, HBV-related complications still cause hundreds of thousands of deaths annually [1]. To mitigate the risk of HBV-related liver disease, it is crucial to monitor treatment effectiveness, viral activity, and disease progression. Biomarkers currently used to evaluate hepatitis B, such as HBV DNA, hepatitis B e antigen (HBeAg), and hepatitis B surface antigen (HBsAg), have limitations in accurately predicting disease outcomes and antiviral treatment responses Vachon, Osiowy [2]. The quantification of intrahepatic covalently closed circular DNA (cccDNA) is regarded as the gold standard for assessing HBV replication and activity. Unfortunately, its clinical application as a prognostic biomarker is limited due to the invasive nature of sample collection and the lack of standardized protocols [3]. Recent research suggests that serum HBV RNA levels could serve as a reliable alternative marker for cccDNA transcriptional activity, helping predict whether a patient can safely discontinue medication based on residual cccDNA transcription [4–7]. Full-length pre-genomic RNA (pgRNA) is the primary RNA species detected in the serum of patients with chronic hepatitis B, it has been investigated for its role in chronic hepatitis B management [4, 8–10]. Currently, Reverse Transcription Quantitative Polymerase Chain Reaction (RT-qPCR) with ultrahigh sensitivity is the common clinical method for detecting HBV RNA, but it faces challenges such as high instrument costs, time-consuming procedures, requirement for trained personnel, and susceptibility to contamination leading to false-positive results. Additionally, no standardized method for HBV RNA detection has been established. Thus, there is an urgent need for a simple, interference-resistant, and sensitive approach to accurately detect HBV RNA in serum.

Since the early 2000 s, the field of dynamic DNA nanotechnology has gained significant attention, with researchers focusing on creating devices for synthesizing DNA-based materials [11–13]. The recent emergence of 3D-DNA Walkers has unlocked their functionality, establishing them as powerful tools for signal amplification in various analytical and diagnostic applications [14]. These DNA Walkers have proven effective in detecting a wide range of analytes with high sensitivity, including nucleic acids [15], proteins [16–18], cancer cells [19], and enzymes [20]. Traditionally, 3D DNA Walker rely on the fluorescence of cleaved substrate single-stranded DNA (ssDNA) for final signal recognition. However, this approach requires complex and costly equipment. Furthermore, limitations in sensitivity and throughput reduce its practicality. As a result, there is an urgent need for more advanced sensing methods that provide greater sensitivity and higher throughput in signal recognition.

Nanopore sensors operate by detecting fluctuations in ionic current caused by analyte translocation or binding events within a nanopore. These fluctuations are recorded and analyzed to identify and quantify analytes at the single-molecule level. Nanopore sensing is capable of detecting and measuring a broad range of substrates, including ions [21], small molecules [22], biomolecules such as proteins [23], DNA [24–30], RNA [31], and even whole cells [32, 33], with remarkable sensitivity. Especially for the sensing of ssDNA, numerous studies have indirectly conducted quantitative analysis of target molecules by achieving the sensing of ssDNA substrates [28, 34–36]. Alpha-hemolysin (α-HL) is a commonly used nanopore protein, with the narrowest region within its pore measuring just 1.4 nm, making it highly suitable for sensing studies of ssDNA. Recent research has explored the use of α-HL nanopores in combination with 3D DNA nanomachines for biomarker sensing [18]. However, these studies have not been validated for clinical applications. Therefore, we propose investigating the integration of nanopores and 3D DNA nanomachines to address the challenges in the clinical detection of HBV RNA.

In this study, we developed a novel strategy that combines 3D DNA nanomachines with nanopore sensing for the detection of serum HBV RNA. This integrated approach encompasses DNA amplification, signal conversion, and the recognition of short oligonucleotide signals. By leveraging the amplification capabilities of 3D DNA nanomachines in conjunction with single-molecule nanopore sensing, we achieved a substantial enhancement in detection sensitivity, thereby demonstrating the robustness of this method. As a result, we accurately quantified HBV RNA levels in serum samples, achieving low limits of detection (LOD) and high precision. This 3D DNA nanomachine-nanopore strategy is characterized by its flexibility, reproducibility, and cost-effectiveness. Additionally, through testing 33 clinical samples, we validated the clinical applicability of this method and its potential for guiding decisions on discontinuing antiviral therapy, making it an attractive tool for clinical biomarker detection. Furthermore, in a double-blind clinical trial, we validated the potential of serum HBV RNA as a reliable biomarker for the diagnosis of hepatitis B and its utility in informing clinical decisions regarding the discontinuation of antiviral therapy.

## Results

### Research principle of the combination of 3D DNA nanomachines and nanopore sensing technology

**Fig. 1:**
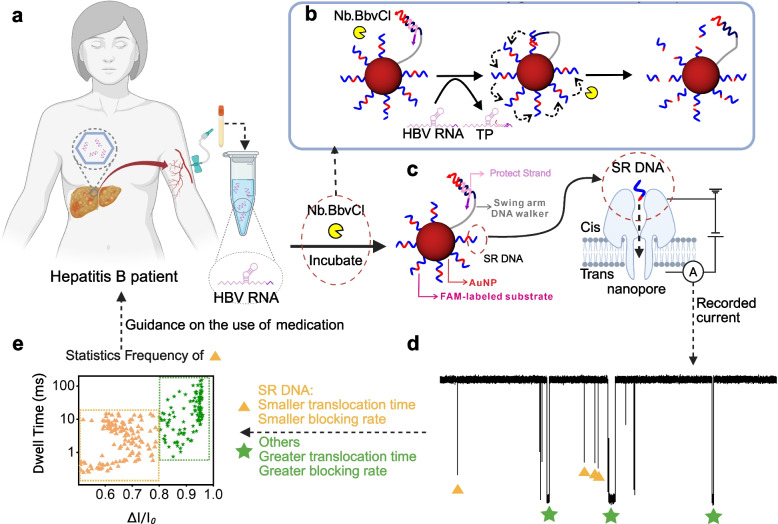
A diagram illustrating the principle of 3D nanomachines combined with nanopore technology for detecting HBV RNA. **a** RNA is extracted from blood samples taken from patients. **b** HBV RNA, an endonuclease enzyme, and the DNA nanomachine are mixed together. The Walker Strand of the nanomachine is activated, and the endonuclease cleaves the SR DNA. **c** The cleaved SR DNA is introduced into the nanopore system, Where it is captured and translocated through the nanopore. **d** Raw current traces of of nucleic acids moving through the nanopores are shown. Yellow triangles indicated signals corresponding to SR DNA, which exhibit lower blocking rates and shorter blocking times. Green pentagons represent signals from interfering substances, which have higher blocking rates and longer blocking times. **e** A scatter plot showing the blocking rate and blocking time of current signals generated by SR DNA translocation through the nanopore. Yellow triangles correspond to SR DNA signals, while green pentagons highlight signals from interfering molecules. Each data point represents a translocation event. TP: Target-Protect Strand. Δ*I*/*I*_*0*_: Blockade ratio. Au NP: Gold Nanoparticle. SR DNA: Signal Report DNA

This study reports a highly sensitive sensor for detecting human HBV RNA, integrating 3D DNA nanomachine-based DNA amplification with nanopore's high sensitivity and specificity for ssDNA signal recognition. Purified HBV RNA from patient peripheral blood was introduced into the pre-prepared DNA nanomachine system (Fig. 1a). In the absence of target HBV RNA, a Protect Strand was designed to prevent activation of the nanomachine's Walker strand (Fig. 1b-c). Upon HBV RNA addition, the Protect Strand competitively bound to its specific recognition site on the viral RNA, dissociating from the Walker Strand's 3'-end and exposing a red-tagged functional domain. This domain hybridized with the complementary red-sequenced ssDNA moiety immobilized on the nanomachine scaffold. Subsequently, a nucleic acid endonuclease recognized the formed dsDNA junction and cleaved the short ssDNA reporter, separating its red and blue fluorescent domains. The released Walker Strand initiated cyclic cleavage by targeting the next ssDNA binding site. Once complete release of the Signal Report (SR) DNA reporter occurred, the solution was transferred to a nanopore detection device. Applying a trans-membrane voltage induced translocation of the cleaved SR DNA through the nanopore. The ultrashort fragment produced distinct blockage amplitude and duration signals during translocation, enabling precise quantification (Fig. 1d-e). These findings establish a foundation for developing platform-based, multi-channel nanopore-integrated detection devices.

### The dual-parameter model based on"blocking rate (0.5–0.8)–blocking time (≤ 10 ms)"can accurately identify SR DNA

**Fig. 2:**
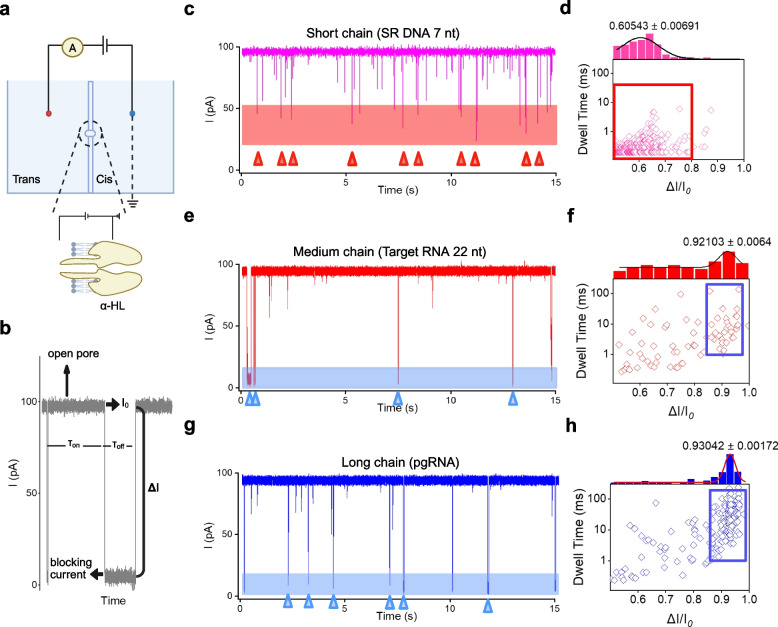
Diagram and analysis of single-stranded nucleic acid movement through nanopores. **a** A diagram showing the setup for electrophysiological experiments with α-HL nanopores inserted. **b** Current trace of the α-HL nanopore in the open state at 100 mV. τ_on_ represents the pore open state, τ_off_ represents the pore closed/blocked state, *I*_*0*_ is the baseline current in the open state, and Δ*I* is the blocking current. **c** A plot of current traces for 7 nucleotide (nt) oligonucleotide SR DNA translocations. **d** The relationship between blocking rate and blocking time for 7 nt SR DNA. **e** A plot of current traces for 22 nt oligonucleotide Target RNA translocations. **f** The relationship between blocking rate and blocking time for 22 nt Target RNA. **g** A plot of current traces for Total RNA from serum samples of patients with clinically positive HBV RNA. **h** The relationship between blocking rate and blocking time for Total RNA from these patients'serum. Triangles below all raw current traces indicate representative events of a single translocation for each single-stranded nucleic acid. Colored regions on each raw current trace represent the blocking rate range for the corresponding nucleic acid translocation events. The recording time for all raw current traces is 15 ms. Each point in the scatter plots represents a translocation event. The upper axis of the scatter plots shows the frequency distribution of blocking rates, with curves fitted using Gaussian functions, and the peak indicates the average blocking rate obtained via Gaussian fitting. The recording time for all scatter plots is 5 min

Before using nanomachines for biosensing, it is crucial to characterize the target signal. This study aims to selectively count ssDNA signals translocating through nanopores to ensure accurate signal detection and counting. The experimental setup for electrophysiological measurements consists of two sample chambers connected by an instrument capable of recording current and applying voltage (Fig. 2a). When α-HL is successfully inserted into the bilayer, the two chambers become connected, enabling ionic current to pass through (Fig. 2b). The nanopore’s conductance of the α-HL nanopore is approximately 0.98 nS, which helps confirm correct insertion (Fig. S1).

Subsequently, nanopores were used to measure electrophysiological signals from three different single-stranded nucleic acids of varying lengths: a 7-base ssDNA, a 22-base target DNA, and total RNA from peripheral blood serum (Fig. 2c, e and g). The signals generated by the translocation of 7-base-length SR DNA through the nanopore mostly fall within the ranges of blocking rate 0.5-0.8 and blocking time 10 ms (red regions in Fig. 2c-d), indicating that SR DNA translocates rapidly through the nanopore with minimal morphological changes. In contrast, the two longer single-stranded nucleic acids exhibit blocking rate and blocking time distributions predominantly centered at > 0.85 and > 1 ms, respectively. Specifically, the blocking times for target RNA typically range from 1 to 100 ms, while pgRNA generally shows even longer blocking times, often exceeding 10 ms (blue regions in Fig. 2e-h). This suggests that longer single-stranded nucleic acids require more time for nanopore translocation and undergo more conformational and structural changes. Furthermore, compared to target RNA, the scatter plots for pgRNA display wider distributions and more frequent blocking times for translocation events. Such variability may be attributed to the presence of other RNAs in serum or enzymatic RNA degradation within the system.

To validate that our"blocking rate-blocking time"dual-parameter identification model can accurately recognize SR DNA in the system, we conducted validation using experimental groups involving nanomachine reactions and positive control groups. First, we introduced RNA from clinical samples into the nanomachine system protected by Protect Strand (Fig. S2a). After complete release of SR DNA, two distinct blocking rates were observed for translocation events. The first signal, with a blocking rate between 0.5 and 0.8 and blocking time typically less than 10 ms, corresponded to SR DNA translocation events (red regions in Fig. S2c and S2 d). The second signal, with a blocking rate > 0.9 and blocking time usually exceeding 10 ms, was interpreted as potential interference from pgRNA or protective probes. Notably, the mean values of the blocking rate distributions fitted for these two signals (0.66 ± 0.01 and 0.95 ± 0.003) showed no significant difference from those obtained by introducing SR DNA and serum RNA alone into the nanopore (0.61 ± 0.01 and 0.93 ± 0.002). Additionally, we performed non-targeted cleavage experiments using specialized nanomachines lacking Protect Strand, where the system theoretically contained only released SR DNA after reaction (Fig. S2b). The translocation events observed predominantly exhibited blocking rates between 0.5 and 0.8 with blocking times shorter than 10 ms (red regions in Fig. S2e-f). Fitting the blocking rate distribution revealed a mean blocking rate of 0.60 ± 0.03, which was highly consistent with the fitting results for SR DNA. These consistent data validate the accuracy of our method for identifying SR DNA. Based on the above analyses, we conclude that the signals relevant for quantitative analysis of SR DNA in this study are specifically distributed within a blocking rate range of 0.5-0.8 and exhibit blocking times ≤ 10 ms.

### 100 mV voltage and pH 4.5 represent the optimal driving force conditions for SR DNA translocation through the nanopore

**Fig. 3:**
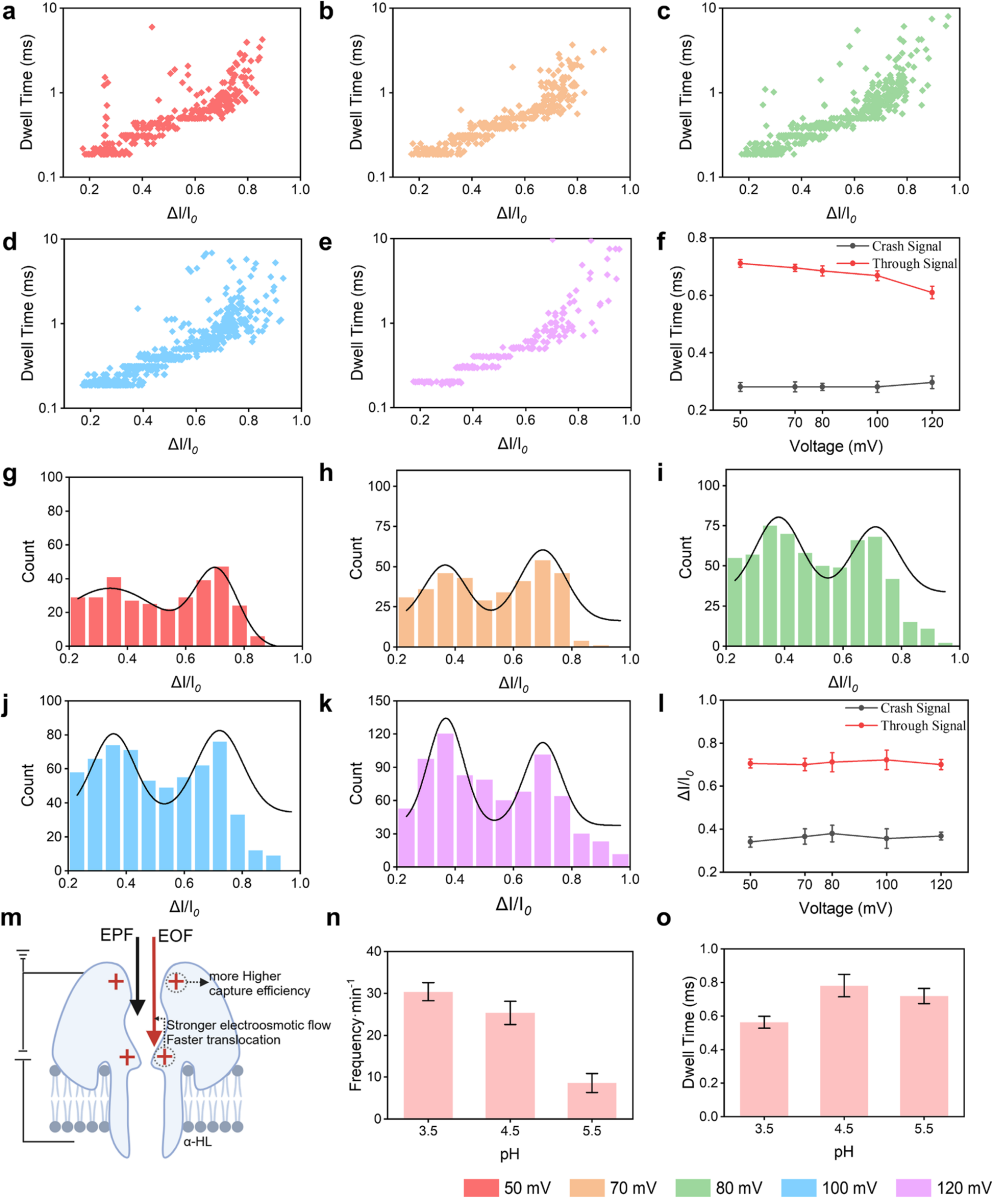
Effects of Different Voltages and pH on SR DNA Nanopore Translocation.** a**-**e**. Scatter plots showing the distribution of blocking rates and blocking events under different voltages. The voltages are color-coded as follows: red for 50 mV, orange for 70 mV, green for 80 mV, blue for 100 mV, and purple for 120 mV. **f**. Trends in the median blocking times of translocation and crash events with varying voltage. **g**-**k**. Distributions of blocking rates for nanopore blocking events at different voltages, with curves obtained via Gaussian fitting. The peak values are the average values of the two blocking rates respectively obtained through Gaussian fitting. **l** The trend in blocking rates of translocation and crash events as a function of voltage. **m**. Schematic Diagram of the Principle of the Influence of Electroosmotic Flow and Electrophoresis on SR DNA Translocation at Different pH Values. **n**. Frequency of translocation events per minute at different pH values. **o** Average blocking time of translocation events per minute at different pH values. Data of n, o were collected at pH 3.5, 4.5, and 5.5. Each data point represents a 15-min statistical average, with error bars indicating the standard deviation from three parallel experiments

The SR DNA released by the nanomachine is only 7 bases in length, and the primary driving forces for ssDNA translocation through the nanopore are electrophoresis and electroosmosis. Excessively high voltage leads to overly strong electrophoretic force, causing SR DNA to translocate too rapidly for the instrument to effectively capture translocation signals. Simultaneously, high voltage may enhance voltage-induced gating effects or background noise. Conversely, insufficient voltage results in low capture efficiency of SR DNA by the nanopore, rendering quantitative analysis challenging. To improve the efficiency of ssDNA translocation through the nanopore and enhance the resolution of target signals, we tested SR DNA translocation events at five voltages ranging from 50 to 120 mV. At each voltage, the fitted curves of blocking rate distributions consistently exhibited two distinct peaks: one corresponding to events with blocking rates below 0.5 and the other to events with blocking rates above 0.5 (Fig. 3a-e and g-k). For events with blocking rates (Δ*I*/*I₀*) < 0.5, blocking times increased with higher voltage (Fig. 3f), indicating that ssDNA undergoes multiple"Crash events"within the nanopore at elevated voltages, prolonging blocking times. These signals were classified as "Crash events" (Fig. S3a). In contrast, for events with blocking rates > 0.5, blocking times decreased as voltage increased (Fig. 3f), suggesting that higher voltages strengthen electrophoretic force, enabling ssDNA to traverse the nanopore more rapidly and generate shorter blocking times. These signals were termed "Translocation events" (Fig. S3b). Notably, the fitted mean values of blocking rates for these two event categories remained essentially constant across different voltages (Fig. 3l), consistent with the fundamental properties of"Crash"and"Translocation"events. As our study requires quantifying HBV RNA indirectly by statistical analysis of SR DNA translocation event frequencies, we must ensure that the blocking rates of the statistical signals exceed 0.5. Additionally, the frequency of SR DNA translocation events per minute clearly increased with voltage (Fig. S3c). To optimize nanopore capture efficiency, an ideal voltage should maximize translocation frequency. However, at 120 mV, excessive nanopore blockage was observed, potentially compromising the accurate quantification of SR DNA. Thus, 100 mV was selected as the optimal voltage, striking a balance between high translocation frequency and manageable nanopore blockage, making it suitable for subsequent experiments.

pH significantly influences nucleic acid translocation events by altering the charge state of amino acid residues within protein nanopores [37, 38]. These changes affect the electroosmotic flow within the channel and the enthalpic contribution to the free energy barrier during ssDNA translocation, potentially inducing conformational changes in ssDNA that accelerate or decelerate its translocation rate (Fig. 3m) [39, 40]. To optimize translocation efficiency, we optimized the electrophysiological buffers under different pH conditions. The frequency of ssDNA translocation per minute increased as the pH decreased from 5.5 to 3.5 (Fig. 3n). This trend may result from a rise in the number of charges at the nanopore openings as the pH decreases. These additional charges likely enhance the nanopore’s attraction to ssDNA, thereby increasing capture efficiency. In terms of translocation duration, there was little variation between pH 5.5 and 4.5, with a slight increase at pH 4.5 (Fig. 3o). This may be due to changes in the enthalpic contribution to the free energy barrier caused by increased charges in the nanopore channel, leading to altered ssDNA conformations and a slower translocation rate. However, at pH 3.5, translocation time decreased again (Fig. 3o). This reduction could be explained by a continued increase in electroosmotic flow within the channel as the charge density inside the nanopore rose further. The combined effects of stronger electroosmotic flow and electrophoretic force likely outweighed the conformational changes that slowed translocation, resulting in faster ssDNA movement and shorter translocation times. The background signal at pH 3.5 increased significantly even without ssDNA (Fig. S4). This rise may be attributed to enhanced electroosmotic flow, which increased ion translocation and potentially improved the sensitivity of ssDNA detection. Considering these observations, we conclude that a symmetric buffer solution with a pH of 4.5 provides the most suitable environment for this protocol, balancing translocation efficiency and background signal.

Based on the dual-parameter model and the above condition optimization, this study constructed an electrophysiological detection system to systematically investigate the quantitative relationship between target molecule concentration and translocation event frequency. When the concentration of SR DNA increased from 10 pM to 10 μM, the frequency of translocation events significantly increased (Fig. S5a). Notably, the frequency of translocation signals generated by SR DNA through the nanopore exhibited an excellent linear relationship with concentration within the range of 100 pM-1 μM, with the detection limit of SR DNA determined to be 4.62 pM (Fig. S5b).

### Characterization demonstrates that the nanomachine was successfully prepared and exhibits a 20-fold amplification capability for HBV RNA

**Fig. 4:**
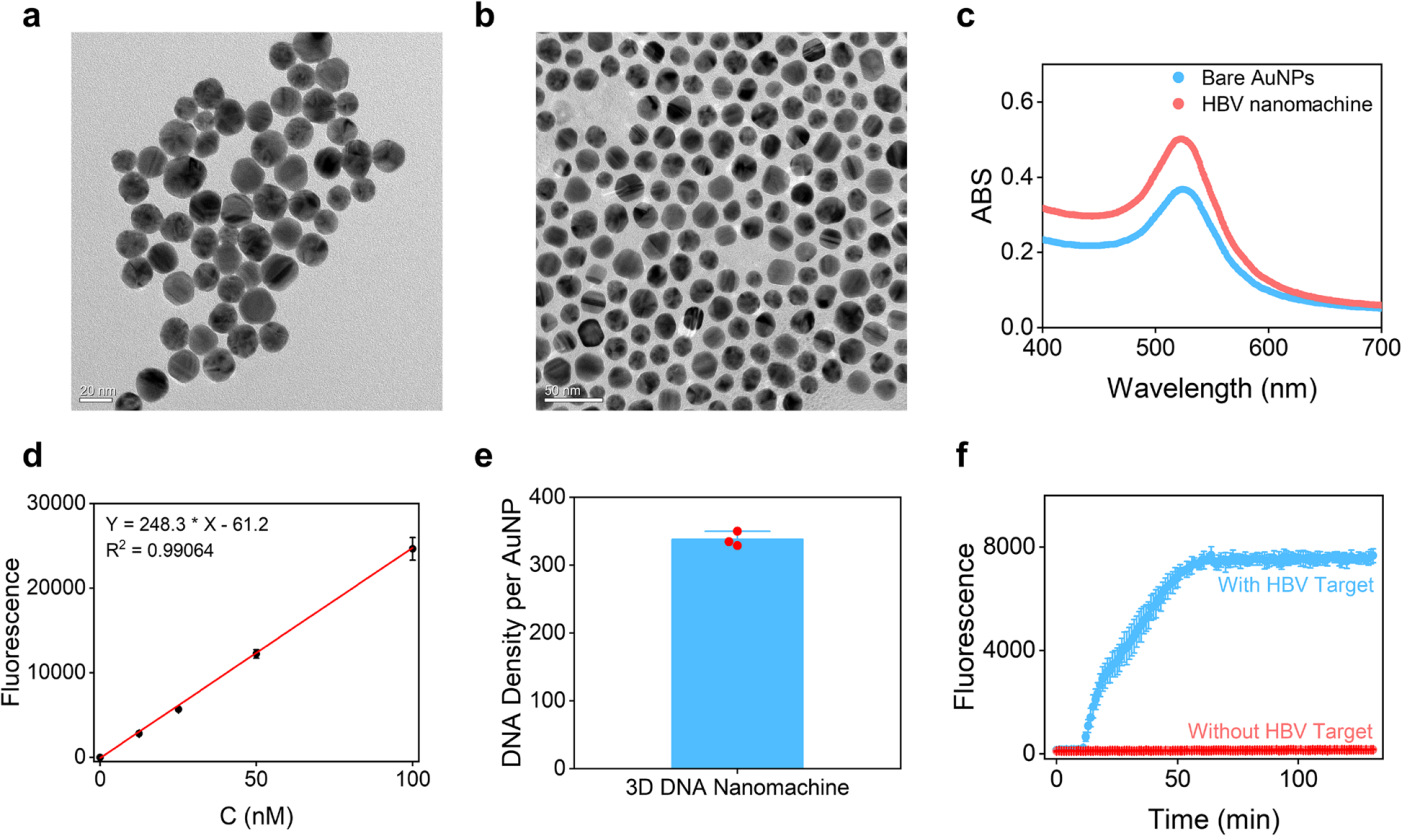
Characterization of nanomachine preparation. **a** TEM characterization of Au NPs. **b** TEM characterization of the prepared nanomachine. **c** UV-vis spectral comparison between Au NPs and the nanomachine. **d** Performance of ssDNA detection via fluorescence. Red lines were obtained by linear fitting. The formula is obtained via linear fitting. **e** Calculated number of functionalized SR DNA on each Au NP. **f** Fluorescence trends with and without target RNA addition for comparison. ABS: Absorbance. C: concentration

To verify the successful fabrication of the nanomachine, transmission electron microscopy (TEM) was used to characterize its particle size. The size distribution of the nanomachines was observed to be concentrated, and were more dispersed compared with the TEM image of Gold Nanoparticles (Au NPs) (Fig. 4a-b), consistent with the design expectations, indicating that steps such as thiolation treatment, annealing, mixed incubation, and ultrasonic dispersion during the preparation process were effectively carried out, successfully constructing a stable nanomachine structure. Additionally, the Ultraviolet-Visible Spectroscopy (UV-Vis) absorption spectrum of the prepared nanomachines exhibited a blue shift of approximately 2 nm at the absorption peak compared to pure Au NPs, confirming that no aggregation occurred in the prepared 3D DNA nanomachines (Fig. 4c). Furthermore, based on the signal peak intensity, the concentration of the prepared nanomachines was roughly estimated to be approximately 2 nM.

During the preparation of the 3D DNA nanomachine, we carefully optimized the stoichiometry of its key components. We determined the molar ratio of Au NPs, SR DNA strands, and DNA Walker strands to be 1:1000:50, which means the ratio of SR DNA strands to DNA Walker strands is 20:1 [15, 41].

Through fluorescence intensity measurements of SR DNA at varying concentrations, we characterized the sensitivity of the fluorescence sensing system for SR DNA, determining LOD of approximately 1 nM (Fig. 4d). Then, by measuring the fluorescence intensity of the labeled SR DNA strands on the surface of the gold nanoparticles, we determined that each 3D DNA nanomachine carried approximately 320 SR DNA strands (Fig. 4e). Combining with the preset molar ratio, we calculated that each gold nanoparticle carried an average of 17 DNA Walker strands. The signal amplification efficiency was evaluated through a series of enzymatic cleavage experiments. The fluorescence-based enzymatic cleavage analysis of the unprotected DNA Walker strand indicated that a single DNA Walker strand immobilized on the surface of the nanoparticle could catalyze the generation of 19 SR strands. This means that the nanomachine has a signal amplification capacity of approximately 20-fold, fully verifying the effectiveness of the design.

To verify whether the 3D DNA nanomachines can respond to HBV RNA, we conducted HBV RNA binding experiments using the nanomachines. The fluorescence intensity of the test group with Target RNA increased continuously over time, whereas the negative control group exhibited a significant difference, with no obvious rise in fluorescence (Fig. 4f). The distinct change in fluorescence signal upon introducing Target RNA molecules indicates that the nanomachines can specifically recognize and bind to Target RNA, triggering the directional movement of DNA Walkers. This process leads to the progressive cleavage of signal probes, releasing a large number of fluorescent signal molecules and enabling highly sensitive signal amplification.

### The 3D DNA nanomachine-nanopore sensor demonstrates excellent sensing performance

**Fig. 5:**
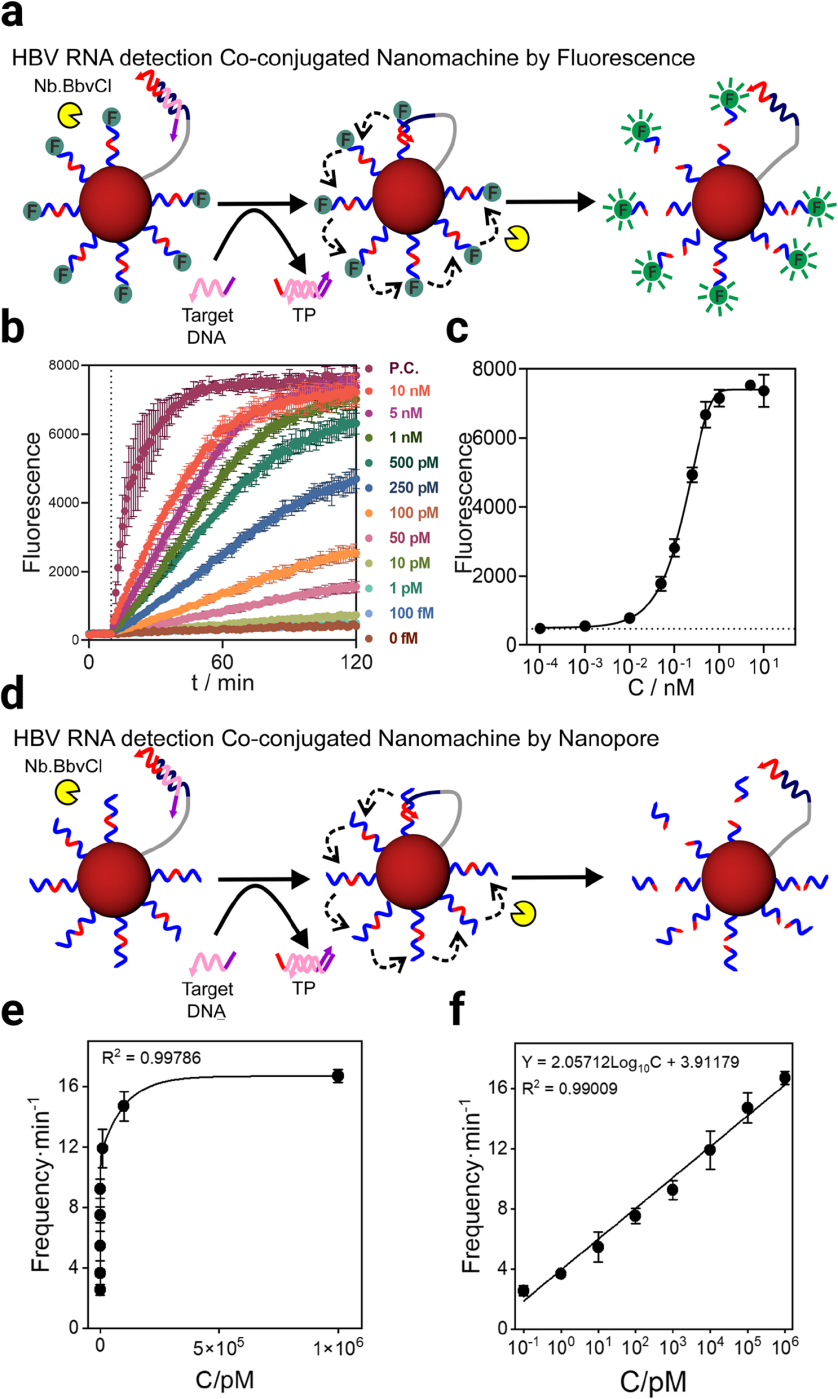
Characterisation of fluorescent and electrophysiological properties of nanomachines. **a** Working principle of fluorescent nanomachine. When no target is added, the fluorescently labeled SR DNA is attached to Au NPs and its fluorescence is quenched. Upon introducing the target, the target binds to the protective strand, exposing the Walker strand, which then binds to the SR DNA on the nanomachine. The Nb.BbvCl endonuclease recognizes the bound double-stranded region and specifically cleaves the SR DNA. The detached SR DNA releases fluorescence again due to dissociation from Au NPs. The target can be indirectly quantified based on the fluorescence intensity. **b** Trend of fluorescence intensity released by the nanomachine with time after adding different concentrations of target DNA. **c** Trend of fluorescence released by the nanomachine with concentration of the target. **d** Working principle of unlabelled nanomachine. The specific scheme is essentially identical to Fig. 5a, except that the SR DNA is unlabeled and the quantification is performed via nanopores. **e**, **f** Translocation events recorded after adding different concentrations of target DNA and adding it to electrophysiological device at the end of the reaction of the nanomachine. events occurring at a frequency of one per minute. The fitting method for e is exponential fitting, and that for f is linear fitting. All error bars represent three completely independent replicate experiments

Subsequently, we verified the performance of the prepared nanomachines using fluorescence-based techniques, conducting concentration analysis of the target through the separation of fluorescent SR DNA from the nanomachines (Fig. 5a). When no target or only a small amount (100 fM) is added, fluorescence shows minimal increase over time (Fig. 5b). In contrast, higher target concentrations cause a significant rise in fluorescence intensity, reaching a maximum approximately 2 h after adding 1 nM of the target. At this point, further increases in target concentration do not substantially enhance the maximum fluorescence intensity, suggesting that the nanomachine activity in solution is nearly complete. Additionally, the time to reach peak fluorescence decreases with higher target concentrations, with maximum efficiency achieved approximately 1 h after adding 10 nM of the target. Finally, we evaluated the fluorescence sensing performance of the nanomachines, with the limit of detection (LOD) being approximately 10 pM, which fails to meet clinical detection requirements (Fig. 5c).

To effectively integrate nanomachines with nanopores, we optimized the key parameters during both the nanomachine reaction and nanopore sensing processes to enhance the amplification efficiency of the nanomachine and the capture efficiency of SR DNA by the nanopore. The optimizations included the volume of reaction solution added to the cis side of the nanopore, the nanomachine's incubation time, and the amount of Nb.BbvCl endonuclease used during incubation. Through systematic testing to balance system stability and reaction speed, we identified the optimal conditions (Fig. S6).

To evaluate the sensitivity of the nanomachine-based sensor using nanopore electrophysiology, we reacted nanomachines with different concentrations of HBV RNA under optimized conditions to isolate unlabeled SR DNA (Fig. 5d). By quantifying SR DNA translocation through the nanopore, we found that the frequency of translocation events significantly increased with rising target concentration (Fig. 5e). The data was also linearly fitted (Fig. 5f), the relationship between event frequency (*f*) and target concentration (C) is described by the equation:

*f* = 2.06 log_10_*C* + 3.91 (*R*^2^ = 0.99).

Moreover, the linear fitting results demonstrate that the sensing scheme in this study exhibits excellent performance for detecting HBV Target RNA, with a linear detection range of 0.1 pM to 1 μM and a detection limit of approximately 12.5 fM. The sensing performance with this sensitivity is capable of meeting clinical detection requirements.

### The sensor exhibits excellent clinical applicability and specificity

**Fig. 6:**
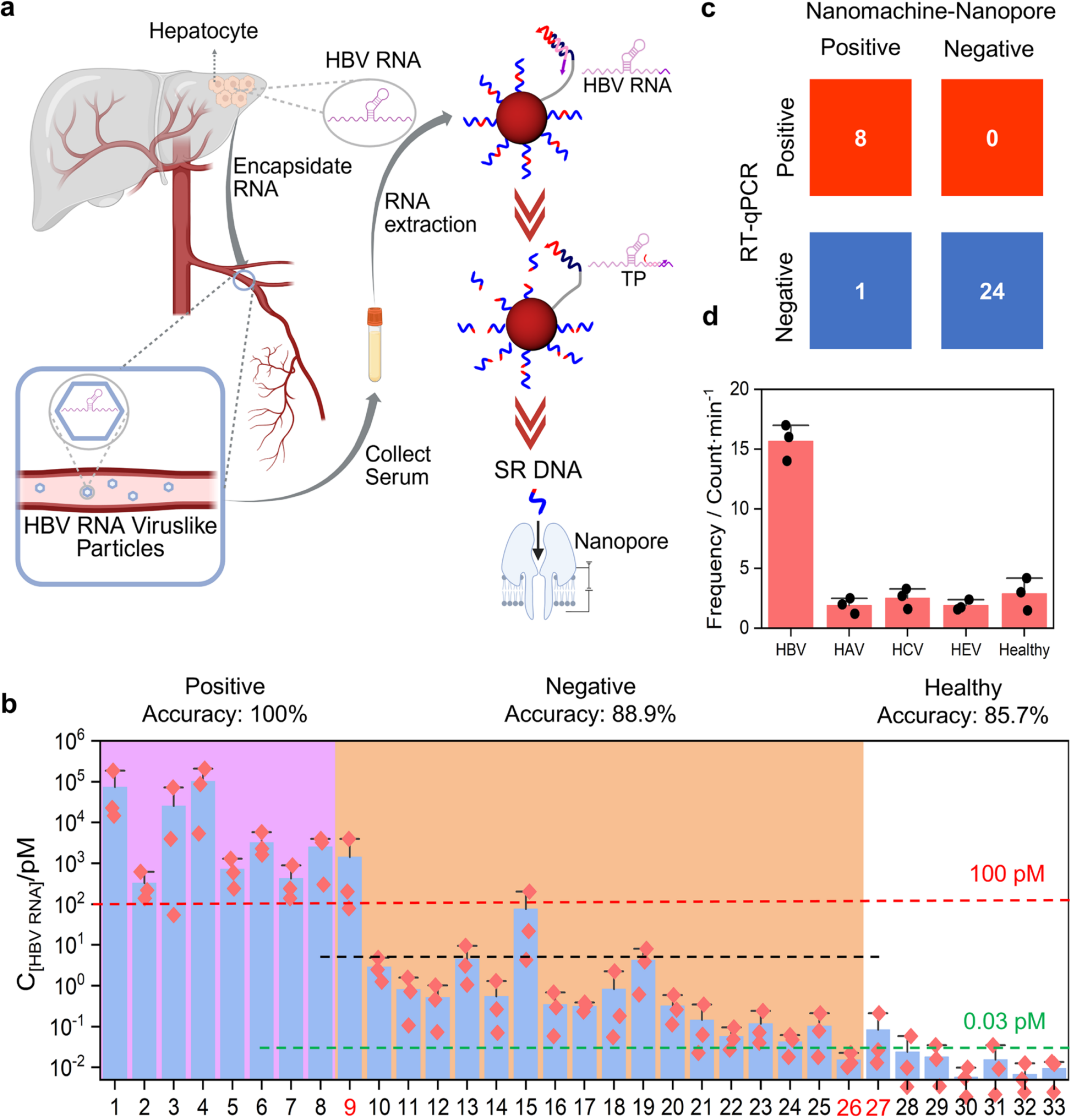
Principles and results of clinical sample testing. **a** Schematic of the detection method for clinical serum samples. Clinically collected peripheral blood serum is subjected to RNA extraction and purification. The obtained RNA is then added to an unlabeled nanomachine reaction system, followed by enzymatic digestion to isolate SR DNA. The isolated SR DNA is quantified by analyzing the nanopore translocation frequency. **b** Detection results of the nanomachine-nanopore assay for 33 clinical serum samples. Different background colors represent the detection results of clinical RT-qPCR: purple (#1-#8) for positive samples, orange (#9-#26) for negative samples, and white (#27-#33) for healthy individuals. The red line indicates 100 pM, where concentrations above this threshold are identified as positive patients by this method, while those below represent negative patients and healthy individuals. The green line at 0.03 pM separates patients from healthy individuals. The black line is used by this method to distinguish between patients requiring continued medication and those eligible for drug discontinuation. Samples labeled in red are misdetected samples by this method. **c**. Heat map of sensitivity and accuracy comparing this method with the RT-qPCR method. **d**. Results of nanopore translocation frequency for HBV, HAV, HCV, HEV, and normal human samples used by this method

To validate the clinical applicability of our method, we tested clinical serum samples from 26 HBV-infected-patients (#1-#26) and 7 healthy-participants (#27-#33) under double-blind conditions. Overall, we extracted RNA from clinically collected samples and added it to the nanomachine-nanopore sensing system for analysis (Fig. 6a). Using the linear relationship between translocation frequency and target concentration, we determined the HBV RNA concentration for each sample (Fig. S7, Fig. 6b). We classified all samples into positive cases, negative cases, and healthy individuals using concentrations of 100 pM and 0.03 pM, where negative cases refer to serum samples from patients undergoing clinical treatment but with negative HBV RNA concentration results.

These results were subsequently compared with the RT-qPCR detection results of HBV RNA in clinical serum samples (Table S2). Through comparison, the results of clinical RT-qPCR are consistent with those of our method for the #1-#8 positive cases. For negative patients, our method misdetects sample #9 and #26. Sample #9 is misdetected as a false positive, and sample #26 with a negative result is misdetected as a healthy individual. However, since the clinical test results for cases after #9 are all negative, the detection result of sample #26 by our method is actually consistent with the clinical RT-qPCR result. The same applies to sample #27, where our method detects a healthy individual as a negative patient. Overall, for the 33 samples, the overall accuracy of our method compared with RT-qPCR was 97%, including a 100% detection rate for positive samples and 96% accuracy for negative samples (Fig. 6c).

In the pathological reports of 18 clinical HBV RNA negative hepatitis B patients (#9-#26), we obtained results including clinical HBsAg levels, HBV DNA levels, and diagnostic outcomes based on multi-marker analysis, as shown in Table S2. These 18 cases were classified into two categories: #9-#20 requiring continued antiviral therapy and #21-#26 eligible for discontinuation of medication. The decision to discontinue treatment was primarily based on the negative results of multiple tested biomarkers and the detection results of other unmeasured biomarkers not available to us. When compared with the HBV RNA detection results of our method, there was a clear demarcation between these two patient groups, where cases above the black dashed line were identified as requiring medication, while those below the line were classified as eligible for discontinuation of medication (Fig. 6b). These results were fully aligned with clinical diagnoses, achieving 100% accuracy. Overall, for patients undergoing treatment who need to assess whether to discontinue medication, this method can provide accurate treatment decisions.

Additionally, we used this nanomachine to detect clinical serum samples from HBV, Hepatitis A Virus (HAV), Hepatitis C Virus (HCV), Hepatitis E Virus (HEV) patients and healthy individuals respectively, to investigate the specificity of the method (Fig. 6d). Results showed that HBV samples exhibited significant differences from other non-HBV samples.

Overall, this method can detect HBV RNA in clinical HBV patients, with results largely consistent with RT-qPCR and an accuracy rate of 97%. Additionally, it achieves 100% accuracy in determining medication continuation or discontinuation for treatment-phase patients, while exhibiting excellent specificity.

## Discussion

This study, building on our team’s prior research, developed a highly sensitive method for detecting HBV RNA in human serum [15, 41–44]. The approach combines three-dimensional DNA nanomachines with nanopore sensing technology. As shown in Fig. 5, the sensing method achieves a detection limit of 12.5 fM. In clinical settings, patient serum HBV RNA concentrations typically exceed 2 pM, making a sub-2 pM detection limit essential for treatment decisions. Our method meets this threshold with sensitivity suitable for clinical use. In contrast to our earlier work, where fM-level detection required 3-4 steps of cascade amplification [43], this study achieves similar sensitivity through nanomachine-mediated nucleic acid amplification alone, demonstrating the efficiency of this amplification strategy. Improved sensitivity also stems from optimizing signal recognition for ssDNA translocation through α-HL nanopores.

The study integrates the strengths of nanomachines and nanopores. Compared with traditional fluorescent nanomachines, the nanomachine-nanopore sensor increases sensitivity by 2-3 orders of magnitude [15, 41]. This improvement is due to nanopores’ better resolution for ssDNA compared to fluorescence-based methods. The method eliminates the need for target RNA to pass through the nanopore, protecting it from degradation by ribonucleases in the electrophysiological environment. Nanopore technology also offers stronger anti-interference capabilities and more stable detection. For RNA targets, the method provides key advantages: low cost, high stability, simple operation, and short reaction times. Unlike RT-qPCR, it skips the reverse transcription step, requiring less time, simpler procedures, and milder temperature conditions, thus enhancing practicality. Since amplification relies on base pairing, modifying the protective strand sequence or adding aptamer probes could extend its use to detect non-RNA or non-nucleic acid targets, positioning it as a versatile signal amplification tool.

Technically, the core focus lies in efficiently linking nanomachine amplification with nanopore sensing, using 7 nt SR DNA as the key connector, where nanomachines accurately recognize HBV RNA for specific target identification. The challenge of SR DNA translocation through nanopores involves isolating characteristic signals from complex serum samples amid interfering substances, with its short length posing a balance problem: excessive voltage speeds up translocation and noise, while insufficient voltage reduces ssDNA capture efficiency. Given limited research on α-HL nanopore sensing of ultrafine oligonucleotides, optimizing electrophysiological conditions like translocation voltage and buffer pH is critical [40]. Though pH affects ssDNA translocation in theory, its impact on 7-nt SR DNA is insignificant due to excessively fast translocation, and prolonging translocation time risks pore blockage and reduced capture efficiency [39]. The optimized pH 4.5 condition was selected based on a comprehensive evaluation of ssDNA blocking rate, blocking time, and background signal noise across three pH conditions. Under these electrophysiological conditions, the sensor achieved extremely low detection limits and could clearly distinguish translocation signals of ssDNA with different lengths. We did not conduct in-depth research on the impact of pH on ssDNA translocation. This represents a limitation of our study, and we plan to address this issue through further exploration in future research. While other optimization conditions may not have reached their optimal states, the current sensor is already sufficient for sensing and recognizing HBV RNA in blood, thus we did not conduct in-depth exploration of these conditions. For subsequent development of multiplexed multi-target detection, nucleic acid primer-probes would need to be redesigned, requiring re-optimization of all experimental conditions. As shown in Fig. 2 and Figure S6, a dual-parameter model of blockage rate and translocation time enabled quantitative analysis of ultrafine ssDNA events by comparing 7-nt oligonucleotides with longer sequences. This differentiation not only identified unique SR DNA translocation signals but also enabled indirect target quantification via nanomachines. Compared with ssDNA fluorescence sensing (Fig. 4d), nanopore sensing improved sensitivity by 2-3 orders of magnitude, highlighting its importance. The combination of three-dimensional DNA nanomachines and the nanopore platform enables clinical HBV RNA detection.

HBV RNA differs from most other HBV biomarkers, which correlate with HBV DNA levels. Serum HBV RNA originates directly from cccDNA, serving as a marker for cccDNA presence and transcriptional activity [2, 4]. As shown in Fig. 6a, HBV RNA is released as virus-like particles after acquiring envelopes, meaning it may persist in patients with undetectable HBV DNA, indicating reactivation risk. Clinically, these patients require continued antiviral therapy, making HBV RNA a key biomarker for treatment discontinuation decisions. Validation using 33 patient samples showed 97% consistency with RT-qPCR, 100% accuracy in predicting treatment needs, and high specificity for distinguishing HBV from other hepatitis viruses. These results suggest the method can assist clinicians in making treatment decisions and reducing clinical workload.

The study has limitations for clinical application. First, while sensitivity and detection limits are met, larger cohort studies are needed to confirm technical reliability. Second, although the method is simpler with portable equipment and low-cost reagents, the 2 h reaction time requires further optimization. High serum protein levels may interfere with nanomachine reactions, necessitating nucleic acid purification and adding steps. Clinically, high-throughput capability is essential; the single-nanopore setup has limited throughput, requiring development of multi-channel devices. Sophisticated data processing tools are also needed to filter noise from serum RNA during nanopore detection. Finally, the investigation of the driving effects of electrophoretic force and electroosmotic flow on ssDNA translocation in this study is solely specific to the SR DNA used herein. For ssDNA of other lengths, deeper mechanistic exploration is required, which will be addressed in our follow-up studies.

## Conclusions

In this study, we integrated a 3D nanomechanical sensor with nanopore sensing technology to develop a highly sensitive assay for the detection of serum HBV RNA in humans. This assay demonstates a broad detection range and a remarkably low detection limit of 12.5 fM, enabling efficient identification of HBV RNA in clinical samples. Furthermore, when compared to clinical RT-qPCR results, our diagnostic approach achieved an accuracy rate of 97%. Additionally, we demonstrated that our strategy predicted the need for medication initiation or discontinuation, with an accuracy of 100%. These results underscore the potential of our method as a valuable adjunct for the clinical diagnosis of HBV RNA and for guiding treatment decisions.

In conclusion, our method offers a simple, rapid, cost-effective, and highly sensitive approach for detecting HBV RNA. It has proven feasible for use with clinical samples, positioning it as a promising supplementary diagnostic tool to aid in the clinical decision-making process for patients requiring medication initiation or cessation.

## Materials and methods

### Chemicals and materials

All DNA and RNA samples were purchased from Sangon Biotech (Shanghai, China) and purified using high-performance liquid chromatography. The sequences and modifications are detailed in Table S1. Gold nanoparticles (20 nm diameter), magnesium chloride hexahydrate (MgCl₂·6H₂O), dithiothreitol (DTT), and 10 × phosphate-buffered saline (PBS) were obtained from Sigma-Aldrich (Oakville, ON, Canada). Nb.BbvCI (10,000 units/mL) and 10 × NEB rCutSmart Buffer were sourced from New England BioLabs (Whitby, ON, Canada). Teflon chambers with a 150 µm pore size were purchased from Warner Instruments (Hamden, CT, USA). Sodium chloride (NaCl) and citric acid were obtained from Sigma-Aldrich (St. Louis, MO, USA). The lipid bilayer material, 1,2-diphytanoyl-sn-glycero-3-phosphocholine (DPhPC), was acquired from Avanti Polar Lipids (Alabaster, AL, USA). Clinical samples were collected from patients and healthy individuals at West China Hospital of Sichuan University. Ultrapure water (18.2 MΩ) was used to prepare buffer solutions for nanopore sensing experiments, including 1 M KCl and 10 mM citric acid (pH 4.5). The RNA extraction kit was purchased from Qiagen (QIAGEN, Germany).

### Instrumentations

Fluorescence emission spectra were measured using a Cytation Hybrid Multi-Mode Reader (BioTek, Vermont, USA). Single-channel current signals were amplified with an Axopatch 200B amplifier (Molecular Devices) and filtered at 2 kHz using a built-in four-pole low-pass Bessel filter. The data were digitized by a Digidata 1550B converter (Molecular Devices) at a sampling rate of 100 kHz. The signals were captured using Clampex 10.2 software, and the collected data were analyzed with Clampfit 10.7 software to determine the block rates and dwell times of blocking events. These results were further analyzed using Origin 2021 software. All electrophysiological recordings were performed at room temperature (23 ± 2 °C) using a buffer solution containing 1 M KCl and 10 mM citric acid (pH 4.5) and applying a voltage of + 100 mV, unless otherwise specified.

### Preparation and characterization of co-conjugated nanomachine

The Signal Reporter (SR) and swing DNA walker (DW) were thiol-modified for attachment to gold nanoparticles (Au NPs). DW was annealed with the protecting strand (T) at a 1:5 molar ratio. Subsequently, substrate strands were mixed with Au NPs at a 1:1000 molar ratio and incubated at room temperature for 24 h. Following incubation, 700 μL of 3 M NaCl solution was added to the mixture, which was sonicated for 10 s and incubated at room temperature for 1 h. This sonication-incubation procedure was repeated five times. The reaction mixture was centrifuged at 13,000 rpm for 15 min, and the pellet was washed with a solution containing 0.5 × PBS, 100 mM NaCl, and 0.05% Tween 20. Three cycles of centrifugation and washing were performed. Finally, the functionalized Au NPs were dispersed in 1 × PBS/100 mM NaCl solution and stored at 4 °C in the dark. Using 45 mM dithiothreitol (DTT), the fluorescently labeled SR DNA on the nanomachine was reductively released. Fluorescence intensity was measured using a multimode microplate reader (SpectraMax i3, Molecular Devices), and the coverage of SR DNA was quantified by using fluorescently labeled free SR DNA as an external standard.

### Real-time monitoring co-conjugated nanomachine on SR DNA

For a typical HBV RNA reaction, a reaction mixture was prepared using the co-conjugated nanomachine SR DNA, containing varying concentrations of HBV RNA target (10 nM to 10 fM), the co-conjugated nanomachine, and 1 × NEB CutSmart buffer. This mixture was then transferred to a 96-well microplate. For experiments involving DNA Walker priming on the track, the walker and designated HBV RNA target were preincubated at 37 °C for 10 min. Following preincubation, 10 units of nicking endonuclease were added, followed by immediate measurement of fluorescence signals. Fluorescence measurements were performed at 37 °C, with signal increments recorded every minute using a multimode microplate reader (excitation/emission: 485 nm/535 nm) to monitor cleavage of the FAM-labeled substrate. For signal normalization, a positive control (co-conjugated nanomachine without the protecting strand, set to 100% signal) and a negative control (co-conjugated nanomachine only) were included.

### Collection of clinical samples

This study was approved by the Biomedical Ethics Committee of West China Hospital, Sichuan University (approval number: 20241192). Patient and healthy human serum samples were obtained from West China Hospital of Sichuan University. The samples were centrifuged at 3500 rpm for 10 min before use. RNA was extracted immediately after receiving the clinical samples to prepare them for subsequent reactions.

### RNA extraction

The RNA extraction steps were strictly performed according to the protocol of the RNA extraction kit. In general, 0.4 mL of serum was taken, and RNA was separated from the serum using chloroform and RNA-Solv reagent. Ethanol and washing buffers were added to wash away impurities through an RNA elution column. Finally, RNA was eluted with 20 μL of DEPC-treated water.

### Nanopore test

Electrophysiological experiments utilized a planar lipid bilayer device comprising a white Delrin cup featuring a 150-µm pore, partitioning the liquid chamber into cis and trans compartments. Cis was grounded, with both compartments equipped with silver/silver chloride electrodes immersed in buffer solution. Each chamber contained 1 mL of electrolyte solution (1 M KCl, 10 mM citric acid, pH 4.5). Lipid bilayers were formed by painting a 40 mg/L solution of 1,2-diphytanoyl-sn-glycero-3-phosphocholine (DPhPC, Avanti Polar Lipids) across the 150-µm pore. α-HL proteins were added to the cis compartment to self-assemble into stable nanopores within the lipid bilayer under applied voltage. Following nanopore formation, pre-incubated samples were introduced to the cis compartment, and recordings were acquired at + 100 mV.

### Statistical analysis

Data processing was performed using Clampfit and Origin software. Using Clampfit, open the raw data file and select the current trajectory within a specific time range. Then, use the "Single—channel Search" function. Set L0 to the baseline current value (*I*_*0*_) and L1 to half of this value (*I*_*0*_/2). This helps identify blockage events and record their blockage time (T) and blockage current (*I*). Next, calculate the blockage rate using the formula Blockade ratio = *I/I*_*0*_.

After that, use Origin software to create a scatter plot. Put the blockage rate on the x-axis and the blockage time on the y-axis. This visualizes the relationship between the two variables. In Origin, perform a Gaussian fit on the blockage rate to find the peak accurately. This identifies the typical blockage rate and allows you to calculate the average blockage rate for the target signal. Also, find the median of the blockage time to better understand the characteristics of the blockage events. This provides essential data for further research.

## Supplementary Information

Supplementary Material 1

## Data Availability

The data supporting this article have been included as part of the Supplementary Information.
