# Supplementary material for: High sensitivity detection of Hepatitis B virus RNA based on 3D-DNA nanomachine and protein nanopore sensing
Source: Mol Biomed. 2025 Aug 8;6:55. doi: 10.1186/s43556-025-00282-7 (PMC12334395; doi:10.1186/s43556-025-00282-7)
Supplement: Supplementary file 1 — Supplementary Material 1 [file 43556_2025_282_MOESM1_ESM.docx]

Supporting information

High sensitivity detection of Hepatitis B virus RNA based on 3D-DNA nanomachine and protein nanopore sensing

Shixin Yan ^1#^, Chuipeng Kong ^2#^, Jiazhe Cheng ^1#^, Zhuoyun Tang ^3#^, Ke Sun ^1^, Shanchuan Chen ^1^, Minghan Li ^1^, Chengyan Tao ^1^, Yue Li ^1^, Yanhua Zhao ^3^, Chuanmin Tao ^3^*, Jia Geng ^1^* and Feng Li ^2^*

AFF1: Department: Department of Laboratory Medicine, State Key Laboratory of Biotherapy and Cancer Center, West China Hospital, Sichuan University and Collaborative Innovation Center, Chengdu 610041, China

AFF2: Key Laboratory of Green Chemistry & Technology of Ministry of Education, College of Chemistry, Sichuan University, Chengdu 610041, China

AFF3: Department: Department of Laboratory Medicine, West China Hospital, Sichuan University, Chengdu 610041, China

* Corresponding Author: taocm@scu.edu.cn (Chuanmin Tao), geng.jia@scu.edu.cn (Jia Geng), windtalker1205@scu.edu.cn (Feng Li).

# These authors contributed equally to this work.

**Results and Discussion**


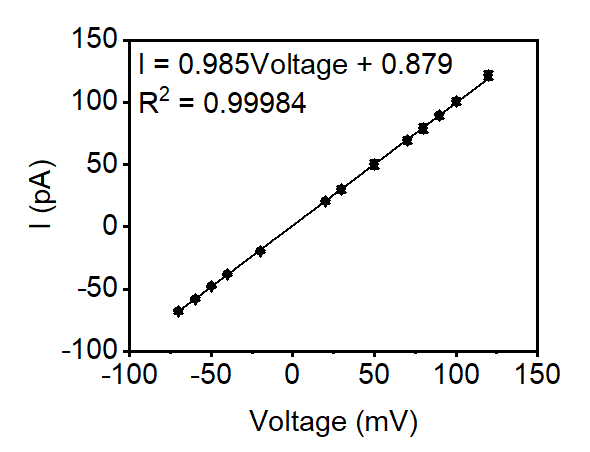


**Figure S1.** Currents measured from α-hemolysin (α-HL) nanopores correctly inserted into planar phospholipid membranes at various applied voltages, with linear fits shown.


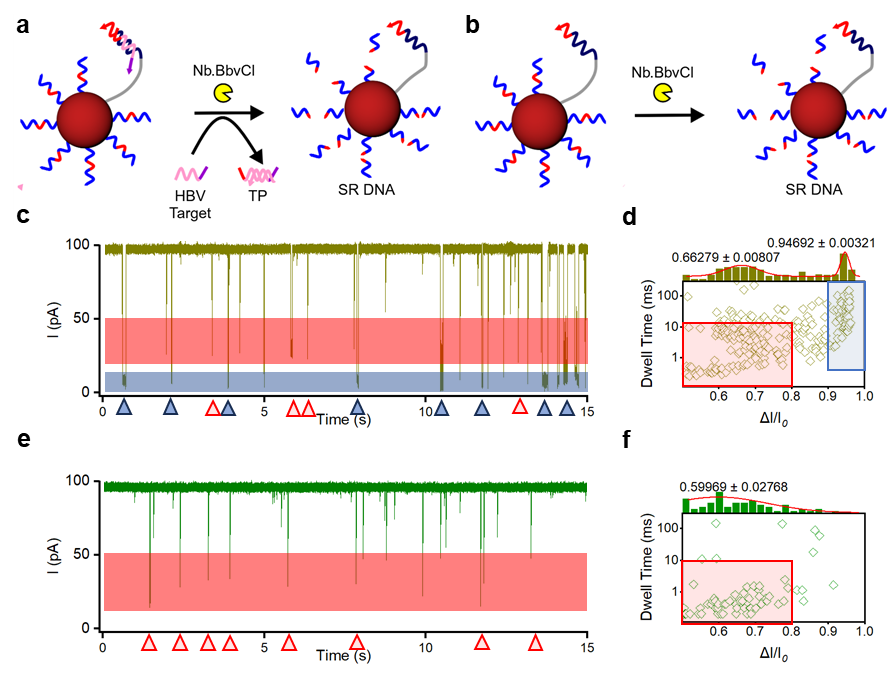


**Figure S2.** 3D nanomachine reaction principles and nanopore translocation signals in the presence and absence of a protection probe. a. Reaction principle of a nanomachine in the presence of a protection probe. b. Reaction principle of a nanomachine in the absence of a protection probe. c. Original current trajectory of the reaction product from the nanomachine with a protection probe translocating through the nanopore, shown over 15 seconds. d. Relationship between blocking rate and blocking time for the reaction product with a protection probe translocating through the nanopore. Each point represents a translocation event. The bar graph shows the frequency distribution of translocation events with blocking rates between 0.5 and 1. The red curve is fitted with a Gaussian function. e. Original current trajectory of the reaction product from the nanomachine without a protection probe translocating through the nanopore, shown over 15 seconds. f. Relationship between blocking rate and blocking time for the reaction product without a protection probe translocating through the nanopore. Each point represents a translocation event. The bar graph shows the frequency distribution of translocation events with blocking rates between 0.5 and 1. The red curve is fitted with a Gaussian function. In the figure, red triangles, red shading, and red boxed events indicate putative translocations of SR DNA. Blue triangles, blue shading, and blue boxed events indicate putative translocations of other DNA or RNA.


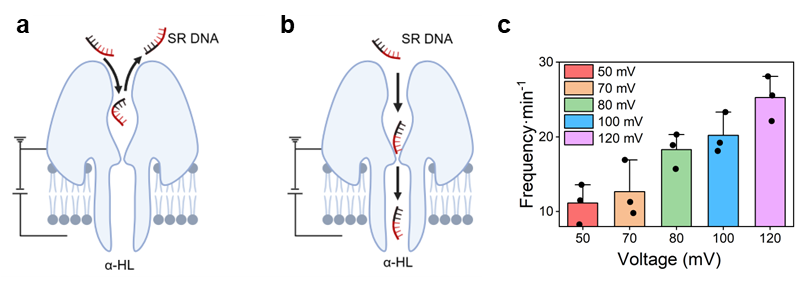


**Figure S3.** Schematic diagrams of collision and translocation events and translocation frequency of ssDNA under different voltages. a. Schematic of "Crash" events. b. Schematic of "Through" events. c. Translocation frequency under different voltages.


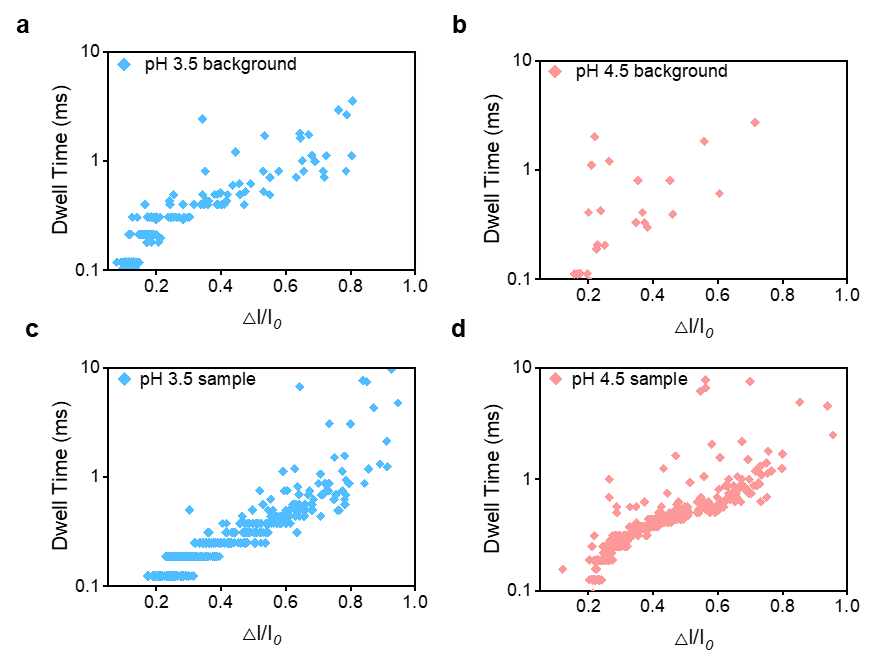


**Figure S4.** Distribution of blocking rate and blocking time for α-HL nanopore background signals and SR DNA events at pH 3.5 and pH 4.5. a. Background signals at pH 3.5. b. Background signals at pH 4.5. c. SR DNA events at pH 3.5. d. SR DNA events at pH 4.5.


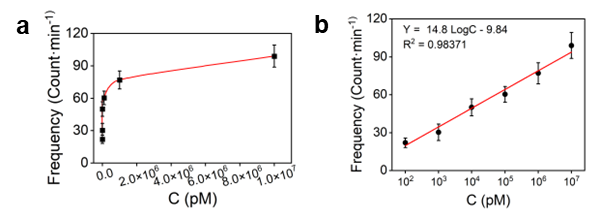


**Figure S5.** Electrophysiological concentration analysis of ssDNA. a. The frequency relationship of ssDNA translocation at different concentrations. b. Linear fitting.


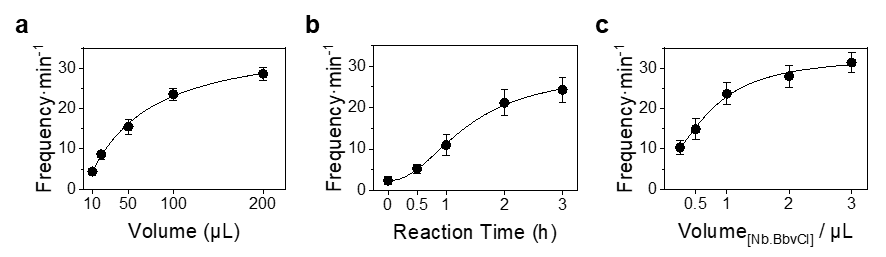


**Figure S6.** Optimization of conditions for sensor performance related to nanomachines. a. Volume of nanomachine reaction solution added to the electrophysiological trans side of the nanopore. b. Optimization of the nanomachine reaction time. c. Optimization of the volume of Nb.BbvCI added to the nanomachine reaction. The vertical axis shows the number of SR DNA translocation events per minute. Error bars represent the standard deviation from three independent replicate experiments.


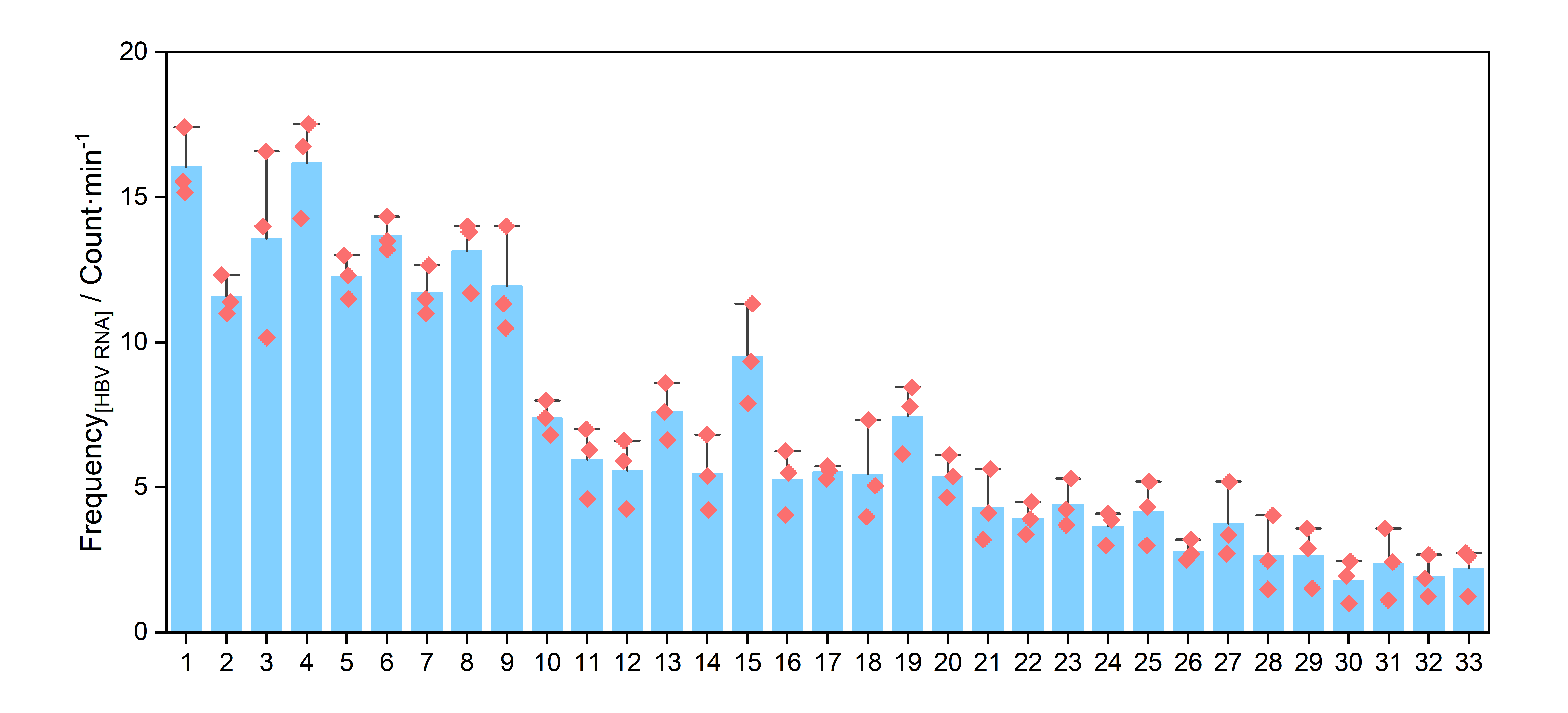


**Figure S7.** Frequency of nanopore translocation events per minute in electrophysiological experiments on 33 clinical samples.

**Table S1. DNA and RNA sequences in this study**

| **DNA name** | **DNA probes for 3D DNA nanomachine** |
| --- | --- |
| Signal Report DNA (SR DNA, Fluorophore) | 5'-SH-T TTT TTT TTT GC^*^T GAG GAT-FAM-3' （^*^cleavage site) |
| SR DNA | 5'-SH-T TTT TTT TTT GC^*^T GAG GAT-3' （*cleavage site) |
| HBV DNA Walker (DW) | 5'-SH-TTT-(T)_55_-TT CAGGCAAGCAATTCTT CCTCAGC |
| HBV Protect Probe | T GAG G AA GAA TTG CTT GCC TGA GTG CAG TAT |
| HBV Target | A UAC UGC AC U CAG GCA AGC AAUU CUU |

**Table S2**. Comparison between Clinical Diagnosis Results of Clinical Samples and Results of This Method

| **Sample ID** | **Gender** | **Age** | **HBV RNA Clinical Result (pM)** | **HBV RNA Nanopore Result (pM)** | **HBsAg Clinical Result (IU/mL)** | **HBV DNA Clinical Result (IU/mL)** | **Clinical Diagnosis Result** |
| --- | --- | --- | --- | --- | --- | --- | --- |
| 1 | male | 41 | 2.05×10^7^ | 73975.48 | 10380 | 903 | positive, require medication treatment |
| 2 | female | 40 | 1.85×10^2^ | 326.15 | 2110 | 38 | positive, require medication treatment |
| 3 | female | 60 | 2.8×10^3^ | 25318.52 | 755 | Below the LOD | positive, require medication treatment |
| 4 | female | 33 | 6.05×10^6^ | 100524.9 | 10760 | Below the LOD | positive, require medication treatment |
| 5 | male | 50 | 6.6×10^2^ | 719.22 | 933 | Below the LOD | positive, require medication treatment |
| 6 | male | 36 | 1.66×10^3^ | 3249.88 | 1933 | Below the LOD | positive, require medication treatment |
| 7 | female | 39 | 5.41×10^2^ | 428.33 | 1494 | Below the LOD | positive, require medication treatment |
| 8 | male | 30 | 9.44×10^2^ | 2506.33 | 5175 | 4550 | positive, require medication treatment |
| 9 | male | 55 | Below the LOD | 1430.38 | 2782 | Below the LOD | Continue medication treatment |
| 10 | female | 53 | Below the LOD | 2.86 | 1176 | Below the LOD | Continue medication treatment |
| 11 | male | 44 | Below the LOD | 0.81 | 10604 | Positive but below the LOD | Continue medication treatment |
| 12 | male | 32 | Below the LOD | 0.51 | 147 | 889 | Continue medication treatment |
| 13 | female | 54 | Below the LOD | 4.55 | 441 | Below the LOD | Continue medication treatment |
| 14 | male | 66 | Below the LOD | 0.54 | 227 | Below the LOD | Continue medication treatment |
| 15 | female | 47 | Below the LOD | 76.31 | 20.8 | Below the LOD | Continue medication treatment |
| 16 | male | 50 | Below the LOD | 0.34 | 25.4 | Positive but below the LOD | Continue medication treatment |
| 17 | female | 50 | Below the LOD | 0.31 | 15.4 | Below the LOD | Continue medication treatment |
| 18 | male | 44 | Below the LOD | 0.83 | 10.9 | Below the LOD | Continue medication treatment |
| 19 | female | 53 | Below the LOD | 4.17 | Below the LOD | 41.7 | Continue medication treatment |
| 20 | male | 49 | Below the LOD | 0.32 | 0.24 | Positive but below the LOD | Continue medication treatment |
| 21 | male | 43 | Below the LOD | 0.14 | Below the LOD | Below the LOD | allow discontinuation of medication |
| 22 | male | 49 | Below the LOD | 0.05 | Below the LOD | Below the LOD | allow discontinuation of medication |
| 23 | male | 49 | Below the LOD | 0.11 | Below the LOD | Below the LOD | allow discontinuation of medication |
| 24 | male | 29 | Below the LOD | 0.04 | Below the LOD | Below the LOD | allow discontinuation of medication |
| 25 | male | 46 | Below the LOD | 0.10 | Below the LOD | Below the LOD | allow discontinuation of medication |
| 26 | female | 44 | Below the LOD | 0.08 | Below the LOD | Below the LOD | allow discontinuation of medication |
| 27 | male | 61 | Below the LOD | 0.02 | Below the LOD | Below the LOD | Healthy |
| 28 | male | 45 | Below the LOD | 0.02 | Below the LOD | Below the LOD | Healthy |
| 29 | male | 50 | Below the LOD | 0.02 | Below the LOD | Below the LOD | Healthy |
| 30 | female | 44 | Below the LOD | 0.01 | Below the LOD | Below the LOD | Healthy |
| 31 | female | 53 | Below the LOD | 0.02 | Below the LOD | Below the LOD | Healthy |
| 32 | male | 46 | Below the LOD | 0.01 | Below the LOD | Below the LOD | Healthy |
| 33 | male | 36 | Below the LOD | 0.01 | Below the LOD | Below the LOD | Healthy |

*All data are truncated to two decimal places.
